# Therapeutic Effects and Molecular Mechanisms of Bioactive Compounds Against Respiratory Diseases: Traditional Chinese Medicine Theory and High-Frequency Use

**DOI:** 10.3389/fphar.2021.734450

**Published:** 2021-08-27

**Authors:** Jing Wang, Qibiao Wu, Lu Ding, Siyu Song, Yaxin Li, Li Shi, Tan Wang, Daqing Zhao, Zeyu Wang, Xiangyan Li

**Affiliations:** ^1^ Department of Respiratory, Changchun University of Chinese Medicine, Changchun, China; ^2^ State Key Laboratory of Quality Research in Chinese Medicines, Faculty of Chinese Medicine, Macau University of Science and Technology, Macau, China; ^3^ College of Integrated Traditional Chinese and Western Medicine, Changchun University of Chinese Medicine, Changchun, China; ^4^ Jilin Ginseng Academy, Key Laboratory of Active Substances and Biological Mechanisms of Ginseng Efficacy, Ministry of Education, Jilin Provincial Key Laboratory of Bio-Macromolecules of Chinese Medicine, Changchun University of Chinese Medicine, Changchun, China; ^5^ Department of Scientific Research, Changchun University of Chinese Medicine, Changchun, China

**Keywords:** Chinese herbal medicines, bioactive compounds, respiratory diseases, therapeutic use, molecular mechanisms of pharmacological action

## Abstract

Respiratory diseases, especially the pandemic of respiratory infectious diseases and refractory chronic lung diseases, remain a key clinical issue and research hot spot due to their high prevalence rates and poor prognosis. In this review, we aimed to summarize the recent advances in the therapeutic effects and molecular mechanisms of key common bioactive compounds from Chinese herbal medicine. Based on the theories of traditional Chinese medicine related to lung diseases, we searched several electronic databases to determine the high-frequency Chinese medicines in clinical application. The active compounds and metabolites from the selected medicines were identified using the Traditional Chinese Medicine Systems Pharmacology Database (TCMSP) by analyzing oral bioavailability and drug similarity index. Then, the pharmacological effects and molecular mechanisms of the selected bioactive compounds in the viral and bacterial infections, inflammation, acute lung injury (ALI), chronic obstructive pulmonary disease (COPD), pulmonary fibrosis, asthma, and lung cancer were summarized. We found that 31 bioactive compounds from the selected 10 common Chinese herbs, such as epigallocatechin-3-gallate (EGCG), kaempferol, isorhamnetin, quercetin, and β-sitosterol, can mainly regulate NF-κB, Nrf2/HO-1, NLRP3, TGF-β/Smad, MAPK, and PI3K/Akt/mTOR pathways to inhibit infection, inflammation, extracellular matrix deposition, and tumor growth in a series of lung-related diseases. This review provides novel perspectives on the preclinical study and clinical application of Chinese herbal medicines and their bioactive compounds against respiratory diseases.

## Introduction

Respiratory diseases include respiratory infectious diseases, asthma, chronic obstructive pulmonary disease (COPD), interstitial pulmonary disease (ILD), and lung cancer. These diseases are characterized by the injuries of bronchial and alveolar tissue to cause respiratory dysfunction and even respiratory failure. Respiratory infectious diseases mainly caused by viruses or bacteria and often contagious, remain a major global public health problem. For example, since the outbreak of coronavirus disease (COVID-19) at the end of 2019, there have been nearly 180 million confirmed cases, including 3.9 million deaths by June 25, 2021 (Mortality et al., 2019; Berlin et al., 2020). Pneumonia is another common respiratory infection, it can lead to hospitalization and death in all age group, and the annual costs exceed $10 billion in the United States and Europe (Global, 2018; Stets et al., 2019). Chronic respiratory diseases such as COPD, ILD, pulmonary fibrosis (PF), and lung cancer, seriously affect human health, these diseases were associated with more than 4 million deaths (7% of all deaths) worldwide in 2017 (Global, 2018). They are induced by long-term exposure to airborne pollutants, tobacco, or kitchen smoke, and their mortality by 18.0% in the last 30 years (Li et al., 2020a). COPD has become the fourth leading cause of death worldwide (Ferkol and Schraufnagel, 2014). Moreover, the quality of life in patients with ILD and IPF is severely affected due to progressive scarring of the lung parenchyma and impairment of pulmonary function (Wollin et al., 2019; Spagnolo et al., 2021). In addition, lung cancer has poor survival and high mortality, and it is the most common cause of cancer-related death worldwide (Siegel et al., 2021). The concern due to the global burden of respiratory diseases, such as the ongoing global pandemic of COVID-19, COPD, and lung cancer, has stimulated research on the treatment and prevention of respiratory diseases. Therefore, the therapeutic effects and molecular mechanisms of potential intervention strategies have become a hot spot for multidisciplinary research.

Traditional Chinese medicine (TCM) has a history of more than 3,000 years and has been used for the prevention and treatment of many respiratory diseases. The ancient medicine books named “Shennong Ben Cao Jing” and “Shanghan Lun” clearly recorded the theories of traditional Chinese medicine, such as reducing phlegm and relieving cough and asthma, and many prescriptions for the prevention and treatment of lung-related diseases. Based on thousands of years of clinical application and the modernization of TCM research, hundreds of Chinese medicines have been shown to be effective in the current clinical applications for treating respiratory infections, asthma, chronic lung diseases, and lung cancer; these effects are based on the therapeutic and improved effects for acute respiratory symptoms and lung dysfunction (Ren et al., 2020; Zhang et al., 2021). Importantly, bioactive compounds or their metabolites from these medicines with high-frequency use, such as saponins, flavonoids, alkaloids, and phenolic acids, are critical for the prevention and treatment of respiratory diseases (Shahidi and Yeo, 2018; Ory et al., 2019; Russo et al., 2020). Currently, the roles of different medicines are summarized in most review, not for potential active components of these medicines for fighting the diseases of respiratory systems. It is necessary to summarize the recent findings regarding the therapeutic effects and molecular mechanisms of bioactive compounds from commonly used Chinese medicines for preventing and treating a series of lung-related diseases. In this review, we first searched English or Chinese electronic databases for clinical studies of TCM against respiratory diseases to identify the Chinese medicines with high-frequency use in the clinical setting. The active components and their metabolites from the selected medicines were identified using the Traditional Chinese Medicine Systems Pharmacology Database (TCMSP) by analyzing oral bioavailability and drug similarity index. Then, the published studies for advanced research of those bioactive compounds after screening in multiple disorders of respiratory system were collected. Finally, we summarized the pharmacological effects and molecular mechanisms of the selected bioactive compounds in the viral and bacterial infections, inflammation, acute lung injury (ALI), COPD, PF, and lung cancer. This review provides new insights into the clinical use of medicinal herbs for the prevention and treatment of respiratory diseases.

## High-Frequency Use of Chinese Medicine and Literature Collection

According to the theories of TCM involving lung-related diseases, we searched Chinese or English electronic databases including CNKI database, Wanfang Data Knowledge Service Platform, VIP Chinese Science and Technology Journal database, PubMed database, and Web of Science database with keywords such as “traditional Chinese medicine,” “Chinese medicine,” or “respiratory diseases.” After the literature retrieval, the Chinese medicines widely used in clinical applications for reducing phlegm (*Morus alba* L., *Moraceae* family, Chinese name: Sangbaipi, peel; *Ginkgo biloba* L., *Ginkgoaceae* family, Chinese name: Baiguo, seed; *Aster tataricus* L.f., *Compositae* family, Chinese name: Ziwan, root) and relieving cough and asthma (*Perilla frutescens* (L.) Britton, *Lamiaceae* family, Chinese name: Suzi, seed; *Tussilago farfara* L., *Compositae* family, Chinese name: Kuandonghua, flower; *Datura metel* L., *Solanaceae* family, Chinese name: Yangjinhua, flower; *Ardisia japonica* (Thunb.) Blume, *Primulaceae* family, Chinese name: Aidicha, leaf; *Lepidium apetalum* Willd., *Brassicaceae* family, Chinese name: Tinglizi, seed; *Eriobotrya japonica* (Thunb.) Lindl., *Rosaceae* family, Chinese name: Pipaye leaf; *Prunus mandshurica* (Maxim.) Koehne., *Rosaceae* family, Chinese name: Kuxingren, seed) were selected.

The effective components and their metabolites of the selected 10 medical plants were searched in the Traditional Chinese Medicine Systems Pharmacology Database (TCMSP, https://old.tcmsp-e.com/index.php, version 2.3). The active compounds of each herb were sorted out by the screening criteria with (oral bioavailability ≥30% and drug-likeness ≥0.18) for the ADME (absorption, distribution, metabolism, and excretion) evaluation system. After sorting, we identified 165 bioactive compounds from these 10 herbs, such as epigallocatechin-3-gallate (EGCG), kaempferol, apigenin, ellagic acid and resveratrol for further analysis. Then, we searched the databases (PubMed, EMBASE, or Web of Science) using the keywords for one of the ingredients from the TCMSP and a type of disease, such as respiratory infection, COVID-19, inflammation, ALI, PF, COPD, asthma, or lung cancer to obtain articles published from January 2000 to May 2021.

**FIGURE 1:**
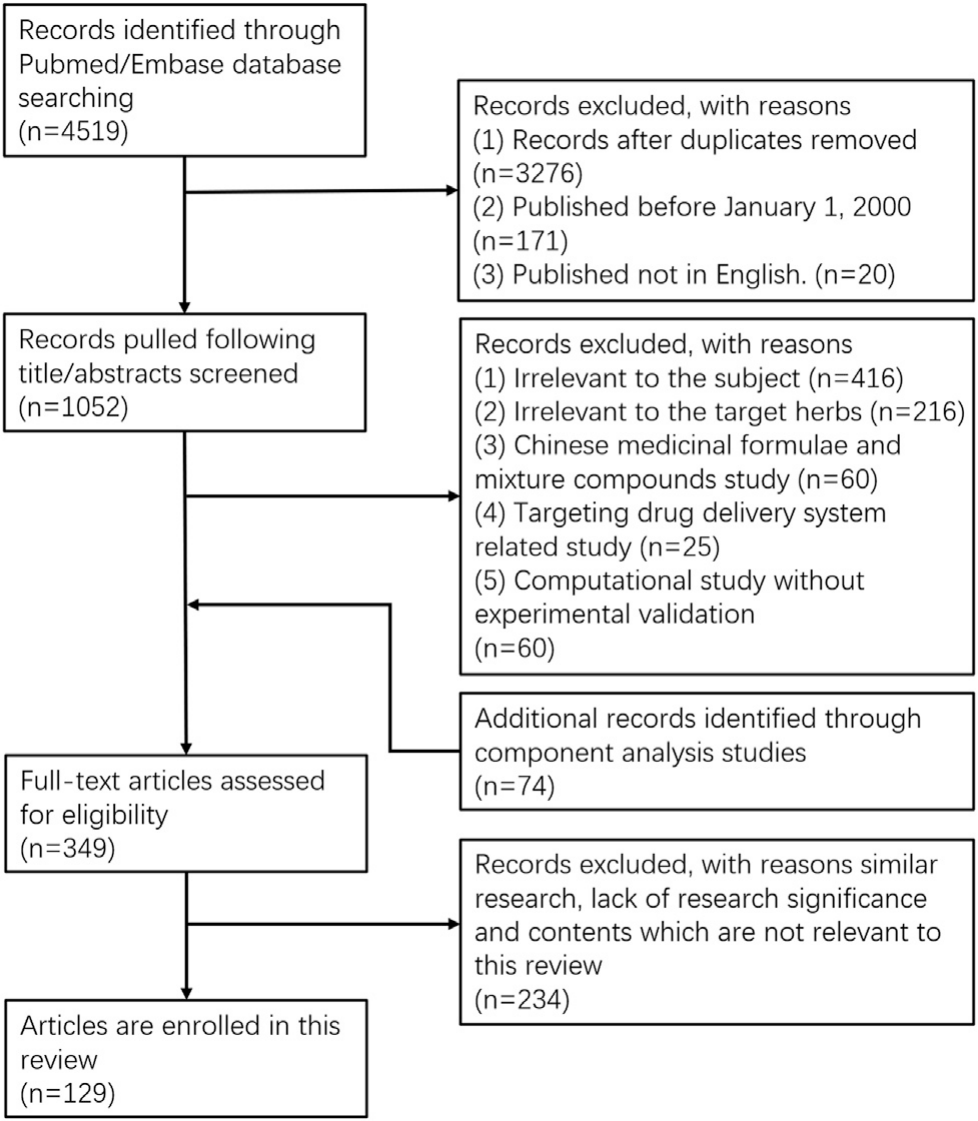
Flow diagram of literature collection methods for bioactive compounds from commonly used of traditional Chinese medicine in clinical applications.

Articles that included both components and disease terms, excluding review articles were identified as reference lists (4,519 articles). Titles and abstracts of all the records were screened to exclude irrelevant studies (duplicates: *n* = 3,276; publication before 2000: *n* = 171, non-English: *n* = 20). We further excluded the irrelevant records for the subject (*n* = 416), target herbs (*n* = 216), Chinese medicinal formulae/mixture compounds (*n* = 60), targeting drug delivery system (*n* = 25), or computational study without experimental validation (*n* = 60). Moreover, 74 reports for component analysis were added to obtain 349 full-text articles for eligibility assessment. Finally, 129 articles for the therapeutic effects and molecular mechanisms of 31 bioactive compounds from the selected 10 herbs were enrolled in the final analysis, after excluding similar studies or those not relevant to our topic of this review (*n* = 234). The detailed flow chart of the published articles collection is shown in Figure 1.

## Therapeutic Effects and Molecular Mechanisms of Bioactive Compounds Against Respiratory Diseases

### Viral and Bacterial Infections

Bacterial and viral infections account for up to 70% of all pathogenic diseases in humans (Smith et al., 2014). Influenza is one of the most prevalent respiratory diseases, and accounts for nearly 5–15% of people all respiratory infections. Although most patients recover, about 0.5 million people die of influenza each year (Petrova and Russell, 2018). The outbreak of COVID-19 has become a global health emergency on a pandemic scale, which has given rise to various studies and developments of antiviral drugs and vaccines. Coronaviruses identify the angiotensin-converting enzyme 2 (ACE2) as the main entry point into the respiratory epithelial cells of the host (Zhou et al., 2020a). Potential targets, including retinoic acid-inducible gene I (RIG-I)/melanoma differentiation-associated gene 5/mitochondrial antiviral signaling/TNF receptor-associated factor 3/interferon regulatory factor 3 (IRF3)/IRF7, and Toll-like receptors (TLRs)/TIR-domain-containing adapter-inducing interferon-β/nuclear factor kappa B (NF-κB)/mitogen-activated protein kinase (MAPK)/activating protein-1 (AP-1) pathways as intercellular sensors have been detected to study translation and budding process of SARS-CoV-1 and MERS viruses infection with SARS-CoV-1 using *in vitro* and *in vivo* models (Stertz et al., 2007), which may cause cellular death, hyperinflammation, and cytokine storm during viral infections (Azkur et al., 2020).

TCM could be a great potential resource for the development of innovative pharmacotherapies against infections. It has been reported that Lianhuaqingwen granules (active ingredients including *Forsythia suspensa* (Thunb.) Vahl, *Lonicera japonica* Thunb., and *Prunus mandshurica* (Maxim.) Koehne., *Rosaceae*) (Jia et al., 2015), Shufeng Jiedu capsule (active ingredients including *Forsythia suspensa* (Thunb.) Vahl, *Strobilanthes cusia* (Nees) Kuntze, and *Bupleurum chinense* DC.) (Liu et al., 2019a), Huoxiang Zhengqi dropping pills (active ingredients including *Pogostemon cablin* (Blanco) Benth., *Platycodon grandiflorus* (Jacq.) A.DC., and *Pinellia ternata* (Thunb.) Makino) (Li et al., 2006) and Haishiyi formula (active ingredients including *Ephedra sinica* Stapf, *Prunus mandshurica* (Maxim.) Koehne.*,* and *Atractylodes macrocephala* Koidz.) can improve clinical symptoms, such as fatigue, cough, and fever, reduce the usage rate of antibiotics, and prevent the progression to severe COVID-19 (Xiao et al., 2020a; Tian et al., 2020; Xia et al., 2021). Currently, canti-COVID-19 agents mainly target SARS-CoV-2 spike receptor-binding domain or ACE2 enzyme activity to block the entry of COVID-19 to the cells. EGCG from *Eriobotrya japonica* (Thunb.) Lindl., and *Ginkgo biloba* L., and isorhamnetin found in *Lepidium apetalum* Willd., *Eriobotrya japonica* (Thunb.) Lindl., *Ginkgo biloba* L., and *Aster tataricus* L.f. exhibit the ability to prevent SARS-CoV-2 from entering into ACE2^+^ cells (Henss et al., 2021; Maiti and Banerjee, 2021; Zhan et al., 2021). Neochlorogenic acid from *Tussilago farfara* L. and Lianhuaqingwen granules inhibit the ACE2 enzyme activity (Chen et al., 2021a). Hesperidin and hyperoside from *Eriobotrya japonica* (Thunb.) Lindl. show antiviral and anti-inflammatory effects against H1N1 virus (Ding et al., 2018; Ling et al., 2020). In H9N2 virus-induced pneumonia, kaempferol inhibits TLR4/Myeloid differentiation factor 88 (MyD88)/NF-κB signaling pathways to reduce the production of inflammatory factors and enhance antioxidant ability (Zhang et al., 2017a). β-sitosterol from eight herbs, such as *Morus alba* L., and *Datura metel* L., inhibits RIG-I and signal transducer and activator of transcription 1 (STAT1) signaling pathway to improve interferon sensitization (Zhou et al., 2020b). As for bacterial infections, it has been reported that benzaldehyde has a good inhibitory effect on a variety of bacteria (Lee et al., 2014a). SARS-CoV-2 spike receptor-binding domain, ACE2, and inflammatory response are essential targets of these bioactive compounds, which may be related to Toll-like receptor and MAPK signaling pathways. Overall, the antiviral and antibacterial effects of these active compounds mentioned above are shown in Table 1.

**TABLE 1:** Summary of effects and mechanisms of bioactive compounds against bacterial and viral infections.

Herbs	Component	Disease/Model	Targets	Mechanism/specific effects	References
*Eriobotrya japonica* (Thunb.) Lindl., *Ginkgo biloba* L.	EGCG	COVID-19/HEK293T cells transfected with the SARS-CoV-2 delta 19 spike gene	SARS-CoV-2 spike receptor-binding domain	Inhibits coronavirus spike proteins	Henss et al. (2021), Maiti and Banerjee (2021)
*Tussilago farfara* L.	Neochlorogenic acid	COVID-19/ACE2 enzyme activity measurement	ACE2	Reduces ACE2 enzyme activity	Chen et al. (2021a)
*Lepidium apetalum* Willd., *Eriobotrya japonica* (Thunb.) Lindl., *Ginkgo biloba* L., *Aster tataricus* L.f.	Isorhamnetin	COVID-19/ACE2 overexpression in HEK293 cells	SARS-CoV-2 spike receptor-binding domain	Inhibits coronavirus spike proteins	Zhan et al. (2021)
*Eriobotrya japonica* (Thunb.) Lindl.	Hesperidin	Virus infection/A rat model using H1N1 virus infection	MAPK signaling pathways	Inhibits pro-inflammatory cytokine production	Ding et al. (2018)
*Eriobotrya japonica* (Thunb.) Lindl.	Hyperoside	Virus infection/H1N1-induced acute lung injury in mice	Toll-like receptor signaling pathway	Reduces cytokine secretion and NF-κB p65 phosphorylation for antiviral and anti-inflammatory effects	Ling et al. (2020)
*Morus alba* L.*, Datura metel* L., and other 6 herbs	Kaempferol	Virus infection/H9N2 influenza virus-induced inflammation *in vivo* and *in vitro*	TLR4/MyD88	Reduces ROS and MPO activity, promotes the production of TNF-α, IL-1β and IL-6; and improves SOD activity	Zhang et al. (2017a)
*Morus alba* L., *Datura metel* L., and other 6 herbs	β-sitosterol	Virus infection/Influenza A virus-induced ALI mice model	Retinoic acid-inducible gene I (RIG-I)	Inhibits RIG-I and STAT1 signaling pathway to improve interferons sensitization	Zhou et al. (2020b)
*Prunus mandshurica* (Maxim.) Koehne, *Perilla frutescens* (L.) Britton	Benzaldehyde	Bacterial infection/16 bacteria and two yeast species	Not available	Not available	Lee et al. (2014a)

### Inflammation and ALI

ALI is common in pulmonary infection, lung contusion, pulmonary embolism, and near-drowning, it can lead to acute respiratory distress syndrome (ARDS) (Suresh et al., 2000). The mortality of ARDS ranges from 35 to 46%, which is higher than mortality of breast cancer or HIV infection (Fan et al., 2018). Patients recovered from ARDS may experience physical, neuropsychiatric, and neurocognitive morbidity that persistently impair their quality of life (Fan et al., 2014). Inflammation, bacterial and viral infections are the most common causes of ALI (Fan and Fan, 2018). The pathogenesis of ALI is believed to be related to inflammation, oxidative stress, cell apoptosis, and hypoxia, involving major cytokines such as tumor necrosis factor-α (TNF-α), interleukin (IL)-6, and IL-1β, IL-9, and IL-8, as well as the chemokines, such as chemokine-2 (CCL-2), monocyte chemotactic factors (MIP), and macrophage chemoattractant protein (MCP). The critical signaling pathways mainly include NF-κB, MAPK, nucleotide-binding oligomerization domain, NOD-like receptor family pyrin domain containing 3 (NLRP3), TLRs, adrenergic receptors, the Janus kinase (JAK)/STAT, and AMP-activated protein kinase (AMPK)- anti-thymocyte globulin (ATG7) signaling pathways (Chang et al., 2018a; Nadeem et al., 2018). The potential targets include of superoxide dismutase (SOD), glutamate-cysteine ligase catalytic subunit (GCLC), NAD(P)H, quinone-1 (NQO1), catalase (CAT), glutathione peroxidase (GSH-Px), and heme oxygenase-1 (HO-1) (Sun et al., 2018; Zhou et al., 2020a). Collectively, intrapulmonary oxidants derived from either activated lung macrophages or oxidant-generating enzymes delivered into the lung are two main pathways of oxidative stress, which can induce ALI and, more seriously, ARDS (Ward, 2010).

The model of ALI is mainly based on the induction by lipopolysaccharide (LPS) in *in vivo* and *in vitro* experiments. Other inflammatory substances and harmful chemicals such as N-methyl-d-aspartate, methamphetamine, and paraquat (PQ) are also used in ALI studies. TNF-α and other cytokines are commonly used in the *in vitro* model construction of ALI. The changes in pulmonary function, lung wet/dry ratios, the morphology of lung tissue, and inflammatory factors in alveolar lavage fluid and serum are generally used to evaluate the inflammatory response. Canonical NF-κB pathway directly induces proinflammatory cytokines such as TNF-α, IL-1β, and IL-6. Subsequently, the activation of IL-1R1 and TNFR1 can make a positive feedback to activate the crucial pathway of inflammation via the NF-κB pathway (Yu et al., 2020a). Ellagic acid, apigenin, EGb761, galangin, isorhamnetin, and kaempferol from *Ginkogo biloba*, *Aster tataricus* L.f., *Eriobotrya japonica* (Thunb.) Lindl., and *Lepidium apetalum* Willd. can reduce the production of inflammatory cytokines and oxidative stress to prevent LPS-induced ALI in mice through the NF-κB pathway (Cornélio Favarin et al., 2013; Huang et al., 2013; Lee et al., 2014b; Shu et al., 2014; Wang et al., 2014; Chi et al., 2016; Li et al., 2016; Luan et al., 2016; Liu et al., 2018a; Qian et al., 2019; Júlio de Souza et al., 2020; Ren et al., 2021). TLR4/MYD88, an upstream player of the NF-κB pathway, mediates the inflammation and ALI. Both processes are ameliorated by ferulic acid and hesperidin from *Aster tataricus* L.f. and *Eriobotrya japonica* (Thunb.) Lindl*.,* which have anti-inflammatory activities and protective effects against ALI by downregulating cytokines and chemokines (Ma et al., 2015; Wu et al., 2021). Furthermore, myeloid differentiation 2 (MD2) and high-mobility group box 1 (HMGB1) are the key targets of hesperidin, through which it can effectively inhibit inflammation during ALI (Liu et al., 2015; Ma et al., 2015). Rutin and moracin M from *Eriobotrya japonica* (Thunb.) Lindl. and *Morus alba* L. improve ALI through a crosstalk of the MAPK and the NF-kB signaling pathways (Yeh et al., 2014; Liu et al., 2015; Ma et al., 2015; Huang et al., 2016; Lee et al., 2016; Ye et al., 2019). The NLRP3 inflammasome processes the interleukin precursors into their mature forms, such as IL-1β and IL-18, which results in inflammation (Afonina et al., 2017). The bioactive components from *Prunus mandshurica* (Maxim.) Koehne, *Eriobotrya japonica* (Thunb.) Lindl., and *Morus alba* L. amygdalin and resveratrol suppress NF-κB activity and ROS production *via* inhibiting NLRP3 inflammasome (Jiang et al., 2016a; Zhang et al., 2017b). SIRT1, the NAD^+^-dependent protein deacetylase, provides “stop signals” for inflammatory and oxidative stress (Jiang et al., 2016a; de Oliveira et al., 2019; Tsai et al., 2019). Resveratrol and oleanolic acid from *Perilla frutescens* (L.) Britton, *Eriobotrya japonica* (Thunb.) Lindl. and *Morus alba* L. reduce PTEN and NF-κB acetylation through the activation of SIRT1 (Peng et al., 2019; Wang et al., 2020a). Quercetin and formononetin from most of the 10 herbs enhance Nrf2/HO-1-mediated cytoprotective effects and prevent LPS-induced lung inflammation (Ma et al., 2013; Takashima et al., 2014; Wang et al., 2018a; Chen et al., 2021b). Luteolin downregulates cytokine and oxidative stress, ICAM-1 through the NF-κB pathway and induces Treg differentiation against ALI (Rungsung et al., 2018; Xie et al., 2021). According to the role of miRNAs in lung inflammation, it has been shown that resveratrol downregulates miR-193a to target transforming growth factor-β2 (TGF-β2), TGFβ receptor (TGFβR3), and death receptor-6 (Alghetaa et al., 2018). Liquiritin from *Prunus mandshurica* (Maxim.) Koehne inhibits the expression of TRPV1 and TRPA1 thereby providing anti-inflammatory and anticough effects (Liu et al., 2020). Taken together, 16 active compounds in 10 herbs have potential roles in inhibiting lung inflammation and injury through NF-κB, MAPK, NLRP3, PI3K/Akt, SIRT1, and HO-1 pathways. More details for the therapeutic effects and molecular mechanism of these compounds against inflammation and ALI are shown in Table 2.

**TABLE 2:** Summary of effects and mechanisms of bioactive compounds against inflammation.

Herbs	Component	Model	Targets	Mechanism/specific effects	References
*Ginkgo biloba* L.	EGb761	LPS-induced ALI	NF-κB pathway	Inhibits NF-κB, phosphorylation of JNK and Akt, TNF-α, interleukin IL-6, macrophage inflammatory protein (MIP)-2, MMP-9, inducible nitric oxide synthase (iNOS), and cyclooxygenase-2 (COX-2)	Huang et al. (2013), Lee et al. (2014b)
*Aster tataricus* L.f.	Apigenin	LPS-induced ALI mice model	NF-κB pathway	Inhibits the expression of NF-κB; reduces IL-6, IL-1β, TNF-α and COX-2	Wang et al. (2014)
*Aster tataricus* L.f.	Apigenin	PQ-induced ALI mice model	NF-κB pathway	Decreases the lung wet/dry ratios and lipid peroxidation, secretion of IL-6, TNF-α and MDA; increases spleen weight, T cell proliferation, secretion of IL-2, glutathione peroxidase (GSH-Px), CAT, and SOD activity	Luan et al. (2016), Liu et al. (2018a)
*Eriobotrya japonica* (Thunb.) Lindl.	Ellagic Acid	LPS-induced ALI mice model	NF-κB pathway	Reduces the vascular permeability changes, the activation of NF-κB and AP-1, and the expression of COX-2, CCL-2, IL-1β, IL-6, IL-10	Cornélio Favarin et al. (2013), Júlio de Souza et al. (2020)
*Aster tataricus* L.f.	Galangin	LPS-induced ALI mice model	NF-κB pathway	Reduces the activation of NF-κB, inflammation and oxidative stress; enhance the expression of HO-1.	Shu et al. (2014)
*Lepidium apetalum* Willd., *Eriobotrya japonica* (Thunb.) Lindl., *Ginkgo biloba* L., *Aster tataricus* L.f.	Isorhamnetin	LPS-induced ALI mice model and TNF-α induced BEAS-2B	NF-κB pathway	Suppresses the phosphorylation of IκBα, NF-κB(p65), ERK and JNK; reduce the level of IL-1β, IL-6, IL-8, TNF-α, and MPO	Chi et al. (2016), Li et al. (2016), Ren et al. (2021)
*Morus alba* L., *Datura metel* L.*,* and other 6 herbs	Kaempferol	LPS-induced ALI mice model	NF-κB pathway	Prevents increased NF-κB and K63-linked polyubiquitination; Reducing lung damage	Qian et al. (2019)
*Aster tataricus* L.f.	Ferulic acid	LPS-induced ALI mice model	TLR4/NF-κB pathway	Reduces the activation of the TLR4 and NF-κB and the secretion of IL-6, IL-1β and TNF-α; ameliorates lung histopathological change	Wu et al. (2021)
*Eriobotrya japonica* (Thunb.) Lindl.	Hesperidin	LPS-induced ALI mice model	NF-κB pathway; MD2; HMGB1	Upregulates the expression of PPAR-γ and inhibits MD2 and HMGB1 to block the interaction between TLR4 and NF-κB; suppresses cytokines and chemokine (TNF-α, IL-6, IL-1β, and MIP-2), the infiltration of macrophages and production of MCP-1	Liu et al. (2015), Ma et al. (2015), Ye et al. (2019)
*Eriobotrya japonica* (Thunb.) Lindl.	Rutin	LPS-induced ALI mice model	MAPK-NF-κB pathway	Inhibits oxidative stress, neutrophil infiltration, VCAM-1, iNOS, and NF-κB activation	Yeh et al. (2014), Huang et al. (2016)
*Morus alba* L.	Moracin M	LPS-induced ALI mice model and alveolar macrophages	MAPK and NF-κB pathways	Downregulates of JNK/c-Jun, NF-κB, IL-6, IL-1β, and iNOS	Lee et al. (2016)
*Prunus mandshurica* (Maxim.) Koehne	Amygdalin	LPS-induced ALI mice model	NLRP3 and NF-κB signaling pathways	Reduces the activation of NF-κB, NLRP3, inflammatory cytokines production (TNF-α, IL-1β, IL-6, and MPO↓) and protect LPS-induced ALI in mice	Zhang et al. (2017b)
*Eriobotrya japonica* (Thunb.) Lindl., *Morus alba* L.	Resveratrol	LPS-induced ALI mice model	NLRP3; PI3K/Akt pathways; Src family kinases (SFKs)	Reduces the NLRP3 inflammasome, ERK and PI3K/Akt pathways; suppresses ROS production (MDA↓ and SOD↑); reduces the level of IL-6, KC, MIP-1α, MIP-2, MCP-1; reduces neutrophil activation and ameliorate lung injury	Jiang et al. (2016a), de Oliveira et al. (2019), Tsai et al. (2019)
*Perilla frutescens* (L.) Britton, *Eriobotrya japonica* (Thunb.) Lindl.	Oleanolic acid	N-methyl-d-aspartate-induced MLE-12 cells apoptosis and lung injury in mice	SIRT1	Activates SIRT1 and reduces the acetylation of NF-κB. Anti-inflammatory (TNF-α, IL-6 and IL-1β) and anti-oxidant (MAD and GSH) functions	Peng et al. (2019)
*Eriobotrya japonica* (Thunb.) Lindl., *Morus alba* L.	Resveratrol	Methamphetamine-induced chronic lung injury	SIRT1/PTEN/p-Akt pathway	Activates SIRT1 and reduces PTEN, phosphorylated Akt. Suppresses ROS levels and LDH leakage, inhibits EMT and the apoptosis	Wang et al. (2020a)
*Morus alba* L., *Datura metel* L., and other 6 herbs	Quercetin	LPS-induced mice model and alveolar macrophage and epithelial cell *in vitro*	heme oxygenase (HO)-1; cAMP	Enhances HO-1-mediated cytoprotective effects for epithelial cell; inhibits the expression of cAMP/Epac, cAMP/PKA, MMP-9, TNF-α, IL-1β, and IL-6; blocks neutrophil recruitment	Takashima et al. (2014), Wang et al. (2018a)
*Ginkgo biloba* L.	Formononetin	LPS-induced ALI mice model	PPARγ; Nrf2/HO-1	Increases PPAR-γ gene expression, Nrf2 and HO-1; reduces hyperoxia and MPO activity; improves SOD activity	Ma et al. (2013), Chen et al. (2021b)
*Eriobotrya japonica* (Thunb.) Lindl., *Aster tataricus* L.f.	Luteolin	ALI mouse model with cecal ligation puncture (CLP)	NF-κB; Treg differentiation	Downregulats IL-1β, IL-6, IL-17A, iNOS, MPO, ICAM-1, and NF-κB; induces Treg differentiation, and increases IL-10 to promote the polarization of M2 macrophages	Rungsung et al. (2018), Xie et al. (2021)
*Eriobotrya japonica* (Thunb.) Lindl., *Morus alba* L.	Resveratrol	Staphylococcal enterotoxin B-exposured mice model	miR-193a	Down-regulates miR-193a targeting TGFβ2, TGFβR3 and death receptor-6; activates apoptotic pathways and promotes anti-inflammatory activities	Alghetaa et al. (2018)
*Prunus mandshurica* (Maxim.) Koehne	Liquiritin	LPS-induced ALI mice model	TRPV1 and TRPA1	Inhibits the expression of TRPV1 and TRPA1; suppresses NF-κB pathway; anti-inflammatory and anti-coughing	Liu et al. (2020)

### Chronic Obstructive Pulmonary Disease

The pathogenesis of COPD is related to chronic inflammation, oxidative stress, cellular senescence, corticosteroid resistance, cell apoptosis, and changes in pulmonary histology and functions. The proinflammatory cytokines and chemokines (TNF-α, IL-1, IL-6, and IL-8), the signaling pathways (NF-κB and MAPK pathways), and various stress-related molecules (SOD, MDA, GSH) participate in the different pathological stages of COPD (Yang et al., 2020). IL-8 recruits neutrophils and secretes several neutrophil elastases and metalloproteases, e.g., MMP-9, which results in alveolar destruction. GM-CSF and IL-6 contribute to the increase in airway smooth muscle mass and proliferation, leading to bronchial obstruction (Knobloch et al., 2018; Jamal Jameel et al., 2021). Human airway smooth muscle cells (HASMCs) contributing to the secretion of cytokines and chemokines are related to non-type 2 airway inflammation and remodeling processes in COPD (Knobloch et al., 2013; Knobloch et al., 2016; Knobloch et al., 2019). Exposure to gases from cigarette smoking and inhaled particles such as PM2.5 are two archetypical inducing factors of COPD, which means that cigarette smoke and PM2.5 are commonly used for establishing *in vivo* and *in vitro* models of COPD (Rabe and Watz, 2017). Many studies have shown that multiple herbs, such as *Tussilago farfara* L., *Eriobotrya japonica* (Thunb.) Lindl., and *Morus alba* L. can inhibit the progression of COPD. Tussilagone and EGCG from the herbs mentioned above enhance the antiproliferative activity through the inhibition of the NF-κB pathway (Choi et al., 2018; Lakshmi et al., 2020). Amygdalin ameliorates the process of epithelial-mesenchymal transition (EMT) through the TGF-β/Smad pathway in cigarette smoke-exposed BEAS-2B cell line and mice model (Wang et al., 2019). Ursolic acid attenuates emphysema and enhances airway remodeling via unfolded protein response (UPR) signaling pathways (Lin et al., 2019; Li et al., 2020b). Liquiritin can reduce pulmonary inflammation by targeting the TGF-β pathway (Guan et al., 2012). Resveratrol inhibits the autophagic process and decreases IL-1β production by inactivation of NLRP3 inflammasome (Ding et al., 2019) or regulation of p53 destabilization (Navarro et al., 2017). Alveolar macrophages are important immune and inflammatory regulatory cells in the lung tissue (Gerlach et al., 2015). Resveratrol reduces the expression of MMP-9, GM-CSF and inflammatory mediators including IL-6, IL-8, and MCP-1 in alveolar macrophages under the stimulation of different harmful substances (Culpitt et al., 2003; Knobloch et al., 2011). Other reports have shown that resveratrol inhibits cytokines and chemokines (CCL-2, IL-6, IL-8) and ameliorates bronchial obstruction-related secretory proteins (GM-CSF and VEGF) in HASMCs from smokers and COPD patients. Similar to the findings against inflammation and ALI, SIRT1 and p38 MAPK are regarded as therapeutic targets of resveratrol in lipoteichoic acid (LTA)- or TNF-α-stimulated HASMC models (Knobloch et al., 2010; Knobloch et al., 2014). The abovementioned therapeutic effects and mechanisms of resveratrol have also been demonstrated in animal models (Chen et al., 2016; Wang et al., 2017a). Together, these findings suggest that six main compounds can regulate NF-κB, UPR, TGF-β, MAPK and SIRT1 pathways to inhibit COPD in different cell and animal models (Table 3).

**TABLE 3:** Summary of effects and mechanisms of bioactive compounds against COPD.

Herbs	Component	Model	Targets	Mechanism/specific effects	References
*Tussilago farfara* L.	Tussilagone	EGF or PMA-induced MUC5AC mucin gene expression and production from NCI-H292 cells	NF-κB pathway and MUC5AC mucin gene	Down-regulates MUC5AC protein, phosphorylation of kappa B kinase (IKK), IκBα, and NF-κB p65;	Choi et al. (2018)
*Eriobotrya japonica* (Thunb.) Lindl., *Ginkgo biloba* L.	EGCG	Cigarette smoke extract-induced normal human bronchial epithelial	NF-κB pathway	Reduces the activation of NF-κB; Anti-oxidative and anti-inflammatory effects	Lakshmi et al. (2020)
*Prunus mandshurica* (Maxim.) Koehne	Amygdalin	BEAS-2B and mice exposed to cigarette smoke	TGF-β/Smad pathway	Suppresses the expression of TGF-β1 and phosphorylated Smad2/3ameliorated EMT process	Wang et al. (2019)
*Perilla frutescens* (L.) Britton; *Eriobotrya japonica* (Thunb.) Lindl.; *Morus alba* L.	Ursolic Acid	PM2.5-induced COPD in rats; cigarette smoke-induced emphysema in rats	UPR signaling pathways	Reduces the p-Smad2 and p-Smad3 on protein level; attenuates CSE-induced emphysema, airway remodeling, and reduces expression of IL-6, TNF-α	Lin et al. (2019), Li et al. (2020b)
*Prunus mandshurica* (Maxim.) Koehne	Liquiritin	A549 exposed to cigarette smoke extract *in vivo*; ICR mice exposed to cigarette smoke	TGF-β and TNF-α	Reduces pulmonary inflammation (TGF-β↓, TNF-α↓); increases anti-oxidative levels (GSH↑)	Guan et al. (2012)
*Eriobotrya japonica* (Thunb.) Lindl.; *Morus alba* L.	Resveratrol	C57BL/6J mice exposed to ambient PM; PM2.5-induced BEAS-2B cells	NLRP3	Reduces the function of NLRP3 inflammasome; inhibits autophagic process and decreased IL-1β production	Ding et al. (2019)
*Eriobotrya japonica* (Thunb.) Lindl.; *Morus alba* L.	Resveratrol	Prematurely ageing telomerase null (terc^−/−^) mice	p53	Enhances p53 destabilization and the expression of PGC-1α, p-Akt, p-Mdm2, p-PTEN; reduces Bax protein; Slowed aging	Navarro et al. (2017)
*Eriobotrya japonica* (Thunb.) Lindl.; *Morus alba* L.	Resveratrol	IL-1β or cigarette smoke media CSM stimulated macrophages which were isolated from BALF from cigarette smokers and COPD patients	IL-8 and granulocyte macrophage-colony stimulating factor (GM-CSF)	inhibited basal release of IL-8 and GM-CSF	Culpitt et al. (2003)
*Eriobotrya japonica* (Thunb.) Lindl.; *Morus alba* L.	Resveratrol	LPS-induced alveolar macrophages from smokers and COPD patients		Reduces secretory protein MMP-9 and inflammatory mediators including IL-6, IL-8, GM-CSF and MCP-1	Knobloch et al. (2011)
*Eriobotrya japonica* (Thunb.) Lindl.; *Morus alba* L.	Resveratrol	Lipoteichoic acid (LTA) from *Staphylococcus aureus* stimulated HASMCs	SIRT1	Reduces CCL-2, IL-6, IL-8 and GM-CSF through activation of SIRT1 or interaction with class I/II HDACs	Knobloch et al. (2014)
*Eriobotrya japonica* (Thunb.) Lindl.; *Morus alba* L.	Resveratrol	TNF-α stimulated HASMCs	p38 MAPK	Reduces the transcription level of IL-8, GM-CSF, and VEGF by inhibiting P38 MAPK	Knobloch et al. (2010)
*Eriobotrya japonica* (Thunb.) Lindl.; *Morus alba* L.	Resveratrol	Cigarette smoke exposure induced rats model	SIRT1 and PGC-1α	Decreases the MDA, IL-6, IL-8 and increases the SOD by increasing SIRT1 and PGC-1α mRNA expression	Wang et al. (2017a)
*Eriobotrya japonica* (Thunb.) Lindl.; *Morus alba* L.	Resveratrol	LPS and cigarette smoke-induced mouse model	Beclin 1	Attenuats the fibrotic response and mucus hypersecretion; Inhibits IL-17, IL-6, TNF-α, and TGF-β through inhibiting Beclin 1	Chen et al. (2016)

### Pulmonary Fibrosis

It is now clear that many elements of the innate and adaptive immune response participate in the differentiation and activation of fibroblasts. The pathogenesis of PF is related to adaptive and innate immune activation, inflammation, epithelial/endothelial damage, EMT and cell apoptosis. Specifically, the activation of TGF-β or NF-κB pathway is the primary factor driving the progression of PF (Kitani et al., 2003; Wynn and Ramalingam, 2012). Some natural products, such as β-sitosterol, quercetin, ferulic acid, hesperidin, and EGb761 from various herbs, inhibit PF by downregulating TGF-β. β-sitosterol and ferulic acid suppress EMT and reduce extracellular matrix (ECM) through the TGF-β/Smad-dependent signaling pathways (Park et al., 2019; Ali et al., 2021). Quercetin suppresses Akt/mammalian target of rapamycin (mTOR) pathway in TGF-β-mediated responses and reduces fibrotic factors, such as collagen I, collagen III, and IL-6 (Xiao et al., 2020b). Another study has reported that quercetin enhances the expression of caveolin1 (CAV1), the cell membrane lipid raft and a protective factor for PF, to inhibit ligand-induced apoptosis in fibroblasts (Hohmann et al., 2019). For other bioactive compounds, hesperidin and EGb761 improve the progression of PF by mediating the proinflammatory cytokines and apoptosis-related proteins *via* the crosstalk of NF-κB and TGF-β pathways (Zhou et al., 2019; Pan et al., 2020).

Hyperoside inhibits the EMT *via* the regulation of the Akt/GSK3β pathway (Huang et al., 2020). Ellagic acid suppresses ECM accumulation by regulating the Wnt pathway (Li et al., 2021). EGCG reduces the production of cytokines through the Nrf-2/HO-1 pathway (Sriram et al., 2009; You et al., 2014). Galangin and isorhamnetin attenuate EMT and inflammatory damage in bleomycin or TGF-β-induced PF models (Zheng et al., 2019; Wang et al., 2020b). Kaempferol promotes autophagy in the therapeutic effects on PF (Liu et al., 2019b). Resveratrol regulates miR-21/Smad7 to alleviate serious PF symptoms (Wang et al., 2018b). Rosmarinic acid targets miR-19b-3p/MYPT1 to relieve the pulmonary fibrosis caused by radiotherapy (Zhang et al., 2020). Collectively, these results indicate that these bioactive compounds can reduce EMT and ECM deposition to inhibit progressive lung fibrosis by regulating TGF-β, Akt/GSK3β, Nrf-2/HO-1, or microRNA-mediated pathways (Table 4).

**TABLE 4:** Summary of effects and mechanisms of bioactive compounds against PF and IPF.

Herbs	Component	Model	Targets	Mechanism/specific effects	References
*Morus alba* L., *Datura metel* L., and other 6 herbs	β-sitosterol	TGF-β-induced human lung alveolar epithelial cell (PF)	TGF-β1/Snail pathway	Inhibits the expression of Snail and Smad2; suppresses EMT and ECM effect	Park et al. (2019)
*Aster tataricus* L.f.	Ferulic acid	silica-induced PF	TGF-β/Smad pathway	Inhibited TGF-β/Smad pathway (CTGF↓, SLUG↓, α-SMA, EMT↓, Vimentin↓, E-cadherin↑); decreases the expression of inflammatory cytokines, and collagen-I; reduces oxidative stress and EMT; attenuates histology	Ali et al. (2021)
*Morus alba* L., *Datura metel* L., and other 6 herbs	Quercetin	LPS-induced Human embryonic lung fibroblast cells (WI-38) and a trauma-induced rabbit tracheal stenosis model	TGF-β/AKT/mTOR pathway	Downregulates expression of mTOR, AKT, ATG; suppressed fibrotic factors (VEGF, IL-6, TGF-β, COL-1, and COL-3)	Xiao et al. (2020b)
*Eriobotrya japonica* (Thunb.) Lindl.	Hesperidin	Bleomycin-induced PF in rat	TGF-β/Smad and NF-κB pathways	Up-regulates expression of Nrf2 and HO-1; down-regulates protein level of AMPK, NF-κB, IκBα, and PP2C-α and mRNA level of TNF-α, IL-1β, IL-6, collagen-1, TGF-β; reduce collagen deposition	Zhou et al. (2019)
*Ginkgo biloba*	EGb761	Bleomycin-Induced PF in Mice	NF-κB/p65 pathway	Reduces protein level of α-SMA and TGF-ß1, phosphorylated NF-κB (p65), caspase-3, and caspase-9; balance of M1/M2 macrophages and NF-κB (p65)-mediated apoptosis	Pan et al. (2020)
*Morus alba* L., *Datura metel* L., and other 6 herbs.	Quercetin	Bleomycin -induced pulmonary fibrosis in aged mice	Balance of p-AKT and CAV1	Enhances expression of CAV1 and reduces expression of p-AKT; inhibits ligand-induced apoptosis (FasL↓ and TRAIL↓) in fibroblasts	Hohmann et al. (2019)
*Eriobotrya japonica* (Thunb.) Lindl.	Hyperoside	Bleomycin-induced pulmonary fibrosis in mice	AKT/GSK3β pathway	Reduces the levels of MDA, TNF-α, and IL-6 and increases the activity of SOD; inhibits the EMT (E-cadherin↑, N-cadherin↓, vimentin↓, TWIST1↓, and SNAIL1↓) via the downregulation of AKT/GSK3β pathway	Huang et al. (2020)
*Eriobotrya japonica* (Thunb.) Lindl.	Ellagic Acid	Bleomycin-induced PF in mice and isolation of primary pulmonary fibroblasts (PPF)	Wnt signaling pathway	Reverses an increase in pro-fibrotic factors hydroxyproline (HYP), ECM accumulation and promotes autophagy of fibroblast through Wnt signaling pathway (Wnt3a↓, β-catenin↓, p-Erk2↓, p-Akt↓, p-mTOR↓, p62↓, Atg16↑, Beclin1↑, LC3-II/I↑)	Li et al. (2021)
*Eriobotrya japonica* (Thunb.) Lindl., *Ginkgo biloba* L.	EGCG	Bleomycin-induced PF in Wistar rats	Nrf-2/HO-1	Activates the expression of Nrf-2 and its downstream HO-1 and NQO-1; reduces lung index scores and histological changes; suppresses the expression of cytokine (TGF-β1↓, IL-6↓, IL-10↓ and TNF-α↓)	Sriram et al. (2009), You et al. (2014)
*Aster tataricus* L.f.	Galangin	Bleomycin-induced PF in mouse and TGF-β1-induced A549 and NIH/3T3 cells	CD4^+^ and CD8^+^ T cells	Increases in the numbers of CD4^+^ and CD8^+^ T cells; attenuates EMT (α-SMA↓, Vimentin↓, E-cadherin↓) and inflammatory damage	Wang et al. (2020b)
*Lepidium apetalum* Willd., *Eriobotrya japonica* (Thunb.) Lindl., *Ginkgo biloba* L., *Aster tataricus* L.f.	Isorhamnetin	Bleomycin-induced PF in mouse and TGF-β-induced HBECs and A549	PERK signaling	Suppresses the activation of PERK signaling (p-PERK↓, p-eIF2α↓, GRP78↓, CHOP↓); inhibits EMT (α-SMA↓, collagen I↓, Vimentin↓, E-cadherin↑) and fibrotic markers, alleviates lung pathologic abnormalities and collagen deposition	Zheng et al. (2019)
*Morus alba* L., *Datura metel* L., and other 6 herbs.	Kaempferol	Bleomycin-induced PF in mouse and silicosis models	Autophagy	Induces LC3 lipidation; promotes autophagy (p62↑) in the therapeutic effects on silicosis	Liu et al. (2019b)
*Eriobotrya japonica* (Thunb.) Lindl.; *Morus alba* L.	Resveratrol	Bleomycin-induced PF in mice and MRC-5 cells	MiR-21	miR-21 targets Smad7 and reduces the phosphorylation levels of ERK, JNK and p38; Decreases the expression of fibronectin, α-SMA, alleviates serious PF symptoms	Wang et al. (2018b)
*Perilla frutescens* (L.) Britton	Rosmarinic acid	X-ray-induced lung injury	MiR-19b-3p	Attenuates RhoA/Rock signaling through up-regulating miR-19b-3p/MYPT1; relieves the pulmonary fibrosis caused by radiotherapy	Zhang et al. (2020)

### Asthma

Asthma is associated with the activation of IgE-mediated mast cells and eosinophilic inflammation. Inhaled corticosteroids which have a therapeutic effect on allergic reactions and sensitivity of type 2 inflammation, are the cornerstone treatment for asthma. Airway inflammation and remodeling, and airway hyperresponsiveness (AHR) promote the pathogenesis of asthma (Mishra et al., 2018). Naïve CD4 T cells are exposed to antigens and differentiate into various T helper (Th) cell types (e.g., Th1, Th2, Th17). Th2 cells play an important role in disease pathogenesis and progression (Chen and Kolls, 2013; Gaurav and Agrawal, 2013). However, neutrophilic inflammation has also been observed during asthma exacerbations as well asc in severe asthma patients (Ray and Kolls, 2017). Through the literature search, natural products from 10 different medical plants have a good inhibitory effect on the inflammation based on eosinophils and neutrophils in asthma. Unsurprisingly, the dysregulation of the NF-κB and MAPK signaling pathways associated with inflammation and immune response, plays a major role in asthma (Freund-Michel and Frossard, 2008; Zhang et al., 2013). Rosmarinic acid, tussilagone, formononetin, galangin, ellagic acid, and ginkgolide B can downregulate the levels of histamine, ovalbumin (OVA)-specific IgE, Th2 cytokines, and chemokines (IL-4, IL-5, IL-13, CCL5, and CCL11) in serum and bronchial alveolar lavage fluid through the suppression of the NF-κB and MAPK signaling pathways (Chu et al., 2011; Alves et al., 2013; Kim et al., 2013; Zha et al., 2013; Zhou et al., 2014; Liang et al., 2016a; Liang et al., 2016b; Henry et al., 2020; Jin et al., 2020; Yi et al., 2020). EGCG inhibits MMP-9 production, ROS generation, and EMT to reduce airway remodeling by upregulating PTEN (Kim et al., 2006; Yang et al., 2018). Kaempferol ameliorates airway hyperplasia and hypertrophy *via* the Syk-PLCγ and PKCμ-ERK-cPLA2-COX2 and NF-κB signaling pathways (Gong et al., 2012; Shin et al., 2015; Molitorisova et al., 2021). Glabridin, β-sitosterol and quercetin can suppress the level of serum IgE, TNF-α, IL-4, and IL-5, but the mechanism has not been thoroughly explored (Rogerio et al., 2007; Mahajan and Mehta, 2011; Dogan et al., 2020). Luteolin inhibits the inflammatory responses and autophagy via the PI3K/Akt/mTOR pathway (Jang et al., 2017; Wang et al., 2021). Taken together, in asthma, these 10 bioactive compounds can inhibit inflammatory reactions and airway remodeling through the MAPK and NF-κB pathways in OVA-induced animal models (Table 5).

**TABLE 5:** Summary of effects and mechanisms of bioactive compounds against asthma.

Herbs	Component	Model	Targets	Mechanisms/specific effects	References
*Perilla frutescens* (L.) Britton	Rosmarinic acid	OVA-induced asthmatic mice model	MAPK and NF-κB pathway	Inhibits expression of ERK, JNK and p38 phosphorylation, activation of NF-κB, Th2 cytokines and IgE, reduces in AMCase, CCL11, CCR3, Ym2 and E-selectin mRNA expression	Liang et al. (2016a), Liang et al. (2016b)
*Tussilago farfara* L.	Tussilagone	OVA-induced asthmatic guinea pigs and IgE-stimulated RBL-2H3 cells	NF-κB and MAPK pathway	suppresses the phosphorylation of Lyn, Syk, Akt, NF-κB p65, ERK and p38 MAPK; down-regulates the levels of histamine, IgE and IL-6 in the serum	Liang et al. (2016a), Liang et al. (2016b), Jin et al. (2020)
*Ginkgo biloba* L.	Formononetin	OVA-induced asthmatic mice	NF-κB and JNK	Inhibits the activation of NF-κB and JNK; enhances the expression of HO-1; ameliorates collagen deposition and oxidative stress, and increases SOD activity; reduces the expression of IL-4, IL-5, IL-13, Ig E, CCL5, and CCL11	Yi et al. (2020)
*Aster tataricus* L.f.	Galangin	OVA-induced BALB/c mice and TGF-β1 induced ASMCs	MAPK/Akt axis; NF-κB pathway	Downregulates the expression of VCAM-1 and p-p65; promotes IκB degradation; upregulates the expression of PPARγ; reduces eosinophil infiltration, hyperplasia and the expression of IL-4, IL-5, IL-13, IL-17, TNF-α, NO, ROS, EPO, CXCL10 and OVA-specific IgE	Kim et al. (2013), Zha et al. (2013), Henry et al. (2020)
*Ginkgo biloba* L.	Ginkgolide B	OVA-induced BALB/c mice	MAPK pathway	Suppresses the expression of MAPK and p-ERK; inhibits the expression of IL-5 and IL-13	Chu et al. (2011)
*Eriobotrya japonica* (Thunb.) Lindl.	Ellagic acid	OVA-induced mouse asthma model	NF-κB pathway	Inhibited NF-κB activation (p-IκB↓, p- NF-κB p65↓); increases Th2 cytokines and inhibits lung eosinophilic inflammation	Alves et al. (2013), Zhou et al. (2014)
*Eriobotrya japonica* (Thunb.) Lindl., *Ginkgo biloba* L.	EGCG	OVA-induced asthmatic mice and TGF-β1-induced 16HBE cells	PI3K/Akt pathway	Inhibits p-PI3K, p-Akt expression through up-regulating PTEN; inhibits MMP-9 production, ROS generation and EMT (α-SMA↓, E-cadherin↑); reduces airway remodeling	Kim et al. (2006), Yang et al. (2018)
*Morus alba* L., *Datura metel* L., and other 6 herbs.	Kaempferol	Bovine serum albumin and OVA-induced BALB/c mice model	Syk-PLCγ, PKCμ-ERK-cPLA2-COX2 and NF-κB pathway	Decreases the levels of IL-5, IL-13, GM-CSF and TGF-β; ameliorates airway hyperplasia and hypertrophy; blunting eosinophil accumulation via suppressing NF-κB pathway	Gong et al. (2012), Shin et al. (2015), Molitorisova et al. (2021)
*Ginkgo biloba* L.	Glabridin	OVA-induced BALB/c mice model	OVA-specific IgE	Suppresses the level of serum IgE; reduces white blood cells and improves respiratory function	Dogan et al. (2020)
*Morus alba* L., *Datura metel* L., and other 6 herbs.	β-sitosterol	OVA-induced airway inflammation in guinea pigs	cytokine	Suppresses the levels of TNFα, IL-4 and IL-5; Upregulates the tidal volume and downregulates the respiration rate	Mahajan and Mehta, (2011)
*Morus alba* L., *Datura metel* L., and other 6 herbs.	Quercetin	OVA-induced BALB/c mice model	IL-5	Reduces neutrophil counts in blood and IL-5 level	Rogerio et al. (2007)
*Eriobotrya japonica* (Thunb.) Lindl., *Aster tataricus* L.f.	Luteolin	OVA-induced mice model	PI3K/Akt/mTOR pathway	Inhibits the OVA-induced inflammatory responses and autophagy via activating the PI3K/Akt/mTOR pathway and inhibiting the Beclin-1-PI3KC3 protein complex	Jang et al. (2017), Wang et al. (2021)

### Lung Cancer

Lung cancer is the malignant tumor with the highest mortality rate. It causes 1.6 million deaths every year, but treatment can effectively prolong survival and quality of life (Siegel et al., 2021). TCM treatment can effectively improve the quality of life and survival time of patients with advanced lung cancer with or without conventional therapy (Sun et al., 2019; Jiang et al., 2016b; Xu et al., 2011). Active components of TCM participate in the treatment of lung cancer through the regulation of multiple pathways (Table 6). Ursolic acid and β-sitosterol show a good lung cancer-inhibiting effect via the TGF-β/Smad pathway (Ruan et al., 2019; Sundarraj et al., 2012; Wang et al., 2017b). Caffeic acid and sanguinarine enhance the antiproliferative effect of paclitaxel in lung cancer A549 and H1299 cells (Lin et al., 2012). Sanguinarine can target NF-κB pathway-mediated autophagy and mitophagy to block lung cancer progression (Yu et al., 2020b). Meanwhile, the p53 protein is a transcription factor that inhibits cell proliferation or survival, acting as a key tumor suppressor protein (Skoulidis and Heymach, 2019). Loss or mutant of p53 induces lung cancer with shortened latency and increases rapid progression and poor prognosis (Donehower et al., 2019). Natural products such as hyperoside, resveratrol, liquiritin, and formononetin have a good effect on improving the antitumor function of p53 and inducing the apoptosis of tumor cells. Hyperoside increases Caspase-9/Caspase-3 activation to induce apoptosis in *in vitro* and *in vivo* models of A549 and H1975 cells (Liu et al., 2016; Lü, 2016). Resveratrol decreases antiapoptotic factors, Bcl-2 and Bcl-xl and the levels of MMP2, and MMP9 by upregulating the p53/HO-1 pathways against lung cancer (Liu et al., 2010; Rasheduzzaman et al., 2018; Li et al., 2019). Liquiritin decreases the expression levels of PCNA, p-PTEN, caspase family, and PARP (Zhou and Ho, 2014). Formononetin promotes Mcl-1 ubiquitination and degradation *via* Fbw7 to enhance the EGFR-TKI sensitivity (Yang et al., 2014; Yu et al., 2020c). The PI3K/Akt signaling pathway is an important dysregulated pathway in tumorigenesis, which controls lung cancer growth, metabolism, motility, and other key cellular processes (Janku et al., 2018). Isorhamnetin and apigenin inhibit EMT and decrease invasion by inhibiting Akt activation (Chang et al., 2018b; Luo et al., 2019). Moracin N induces autophagy mTOR signaling pathway (Gao et al., 2020). Furthermore, isorhamnetin as a potential application in adjuvant radiotherapy inhibits the activation of NF-κB and increases the expression of IL-13 (Du et al., 2020). Resveratrol and ellagic acid promote lung cancer cell apoptosis *via* the PI3K/Akt signaling pathway (Liu et al., 2018b; Li et al., 2019). Amygdalin downregulates the phosphorylation of Akt to inhibit invasion and migration of H1299 and PA cells (Qian et al., 2015). Inactivation of STAT3 is a target for increasing cisplatin sensitivity in lung cancer treatment, galangin, and laricitrin are STAT3 inhibitors in adjuvant chemotherapy (Chang et al., 2016a; Chang et al., 2016b; Yu et al., 2018). Oleanolic acid enhances mitophagy through the PINK1/Parkin axis in A549 cells (Castrejón-Jiménez et al., 2019). Rosmarinic acid could reverse the cisplatin resistance by inhibiting the expression of P-gp, MDR1, and MAPK pathways and plays a key role in the treatment of non-small cell lung cancer (NSCLC) (Liao et al., 2020). EGCG from *Eriobotrya japonica* (Thunb.) Lindl. and *Ginkgo biloba* L. can suppress the levels of Axl and Tyro three to reduce the resistance to platinum (Kim and Lee, 2014). Ginkgolide B and glabridin from *Ginkgo biloba* L. have inhibitory effects on autophagy and angiogenesis, mediated by Beclin-1 or FAK/Src complex, respectively (Tsai et al., 2011; Wang et al., 2020c). In H1975 cell model, ursolic acid inhibits the Wnt/β-catenin pathway to suppress proliferation and induce apoptosis (Yang et al., 2019). As a cisplatin sensitizing agent, ginkgetin enhances the ferroptosis-mediated disruption of the Nrf2/HO-1 axis (Lou et al., 2021). Kaempferol downregulates Nrf2 and upregulates miR-340 to induce apoptosis and autophagy (Han et al., 2018; Fouzder et al., 2021). As for quercetin, it can target aurora B or miR-16-5p/WEE1 pathways to inhibit lung cancer progression and enhance the radiosensitivity of NSCLC cells (Xingyu et al., 2016; Wang et al., 2020d). Hesperidin exhibits antiproliferative and apoptosis induction effects by regulating the miR-132/ZEB2 signaling pathway (Birsu Cincin et al., 2015; Tan et al., 2020). Luteolin inhibits cell proliferation and induces apoptosis via miR-34a-5p targeting MDM4 and RhoA (Jiang et al., 2018; Masraksa et al., 2020). Taken together, these results demonstrate that these bioactive compounds have anticancer effects by targeting multiple pathways, including NF-κB, p53, TGF-β, or miRNAs (Table 6).

**TABLE 6:** Summary of effects and mechanisms of bioactive compounds against lung cancer.

Herbs	Component	Model	Targets	Mechanism/specific effects	References
*Perilla frutescens* (L.) Britton; *Eriobotrya japonica* (Thunb.) Lindl.; *Morus alba* L.	Ursolic acid	H1975 cells	TGF-β1 signaling pathway	Reduces TGF-β1 pathway to regulate integrin αVβ5 and MMP9 expression; inhibits the cell migration, invasion EMT in H1975 cells	Ruan et al. (2019)
*Morus alba* L., *Datura metel* L., and other 6 herbs.	β-sitosterol	A549, NCI-H1975 and H1299 cells	TGF-β/Smad2/3 pathway	Inactivates TGF-β, Smad2/3 and c-Myc; inhibits autophagy and induced G_0_/G_1_ cell cycle arrest and inhibits cell proliferation	Sundarraj et al. (2012), Wang et al. (2017b)
*Aster tataricus* L.f.	caffeic acid	A549 and H1299 cells	NF-κB pathway	Reduces nuclear translocation of NF-κB p65; sensibilization of paclitaxel; anti-proliferation and apoptosis	Lin et al. (2012)
*Morus alba* L.	Sanguinarine	A549 and THP-1 *in vivo* model	NF-κB pathway	Inhibits p-p65 expression via exosomes; suppresses the expression of TNF-α, IL-6, and CCL-2; induces autophagy and mitophagy	Yu et al. (2020b)
*Eriobotrya japonica* (Thunb.) Lindl.	Hyperoside	A549 and H1975 *in vivo* and *in vitro* model	Caspase-3, p53, and NF-κB signaling pathway	Inhibits NF-κB transcriptional activity, enhances Caspase-9/Caspase-3 activation; induces apoptosis and inhibits proliferation	Liu et al. (2016), Lü (2016)
*Eriobotrya japonica* (Thunb.) Lindl.; *Morus alba* L.	Resveratrol	A549, HCC-15, ASTC-a-1, PC14, H69, and IMR90	p53, PRMT5; HO-1	Decreases the phosphorylated Akt, PRMT5 and NF-κB via upregulation of p53 and HO-1; promotes cancer cell apoptosis (Bcl-2↓, Bcl-xl↓, cyclin D1↓, cyclin E1↓); inhibits invasion (MMP-9↓; MMP-2↓)	Liu et al. (2010), Rasheduzzaman et al. (2018), Li et al. (2019)
*Prunus mandshurica* (Maxim.) Koehne	Liquiritin	A549 cells	p53 and p21	Upregulates p53 and p21; induces apoptotic pathways (p53↑, p21↑; PCNA↓, MDM2↓, p-GSK3β↓, p-Akt↓, p-c-Raf↓, p-PTEN↑; PARP↓, Bcl-2↓, caspase family↑)	Zhou and Ho, (2014)
*Ginkgo biloba* L.	Formononetin	A549 and NCI-H23 cells	p53, EGFR-Akt-Mcl-1 axis	Enhances Mcl-1 ubiquitination via degradation of Fbw7; increases the phosphorylation of p53; promotes the EGFR-TKI sensitivity; induces cell cycle arrest and apoptosis	Yang et al. (2014), Yu et al. (2020c)
*Lepidium apetalum* Willd.*, Eriobotrya japonica* (Thunb.) Lindl., *Ginkgo biloba* L., *Aster tataricus* L.f.	Isorhamnetin	A549 cells	Akt/ERK1/2 and NF-κB pathway	Suppresses the expression of Akt, ERK1/2, IL-13, and NF-κB p65; inhibits EMT, MMP-2 and MMP-9 activity	Luo et al. (2019), Du et al. (2020)
*Aster tataricus* L.f.	Apigenin	A549, CL1–5, HCC827, and H1975 NSCLC *in vitro* and A549 *in vivo* models	CD26/DPPIV	Suppresses the expression of CD26, DPPIV and Akt; modulates EMT (Snail↓, Slug↓) and decreases invasion	Chang et al. (2018b)
*Eriobotrya japonica* (Thunb.) Lindl.	Ellagic acid	A549 cells	PI3K/Akt signaling pathway	Reduces the phosphorylation of PI3K and Akt; suppresses cell proliferation, induces apoptosis (Bax↑, Bcl-2↓, Caspase-3↑, p21↑)	Liu et al. (2018b)
*Morus alba* L.	Moracin N	A549 and PC9 cells	mTOR signaling pathway	Inhibits the expression of p-S6 EGFR; reduces ROS generation, promotes cancer cell autophagy (p-AKT↓, p-mTOR↓) and apoptosis (Bax↑, Bcl-2↓, Caspase-9↑)	Gao et al. (2020)
*Prunus mandshurica* (Maxim.) Koehne	Amygdalin	H1299 and PA cell	Akt and RICTOR	Down-regulates the expression of cell integrin β1/4 and FAK; inhibits the *in vitro* invasion and migration (E-cadherin↑)	Qian et al. (2015)
*Ardisia japonica* (Thunb.) Blume	Laricitrin	A549, CL1-5, and H1395 *in vivo*; LLC cells implanted into C57BL/6	BRAF; STAT3	Inhibits the phosphorylation of STAT3 and expression of IL-10; changes the CD4^+^ T cell phenotype from Th2 to Th1; ameliorates BRAF mutation-induced lung cancer; enhances the DDP sensitivity	Chang et al. (2016a), Chang et al. (2016b)
*Aster tataricus* L.f.	Galangin	A549 and A549/DDP	STAT3	Suppresses the NF-κB, Bcl-2/Bax ratio via inactivating p-STAT3/p65; enhances the DDP sensitivity	Yu et al. (2018)
*Perilla frutescens* (L.) Britton; *Eriobotrya japonica* (Thunb.) Lindl.	Oleanolic acid	A549 cells	PINK1/Parkin axis	Decrease p62 and Nrf2 proteins, induces an ROS production; promotes ROS production and mitophagy (p-AKT↓; p-mTOR↓)	Castrejón-Jiménez et al. (2019)
*Perilla frutescens* (L.) Britton	Rosmarinic acid	A549 cisplatin-resistant cells	MAPK signaling pathway	Inhibits the expression of P-gp and MDR1, enhances p-JNK, p-c-JUN, p21 and p53 expression; DDP resistance reversal agent in NSCLC	Liao et al. (2020)
*Eriobotrya japonica* (Thunb.) Lindl.; *Ginkgo biloba* L.	EGCG	A549 and H460 platinum-resistant cells	Axl, Tyro3	Suppresses the expression of both Axl and Tyro 3 receptor tyrosine kinases; reduces platinum-resistance	Kim and Lee, (2014)
*Ginkgo biloba* L.	Ginkgolide B	A549 and H1975 cells	Beclin-1	Reduces Beclin-1, induces inhibition of NLRP3 and autophagy (Bcl-2↓, PCNA↓, p62↑)	Wang et al. (2020c)
*Ginkgo biloba* L.	Glabridin	A549 cells	FAK/Src complex	Inhibits the FAK/Src complex; suppresses the activation of Akt and RhoA; promotes inhibition of migration, invasion, and angiogenesis	Tsai et al. (2011)
*Morus alba* L.; *Perilla frutescens* (L.) Britton; *Eriobotrya japonica* (Thunb.) Lindl	Ursolic acid	H1975 cells *in vitro* and *in vivo* models	Wnt/β-catenin signaling pathway	Suppresses CT45A2 gene transcription by inhibiting TCF4 and β-catenin; inhibits proliferation and enhances apoptosis of H1975	Yang et al. (2019)
*Ginkgo biloba*	Ginkgetin	A549, NCI-H460, and SPC-A-1 cells and A549 xenograft nude mouse model	Nrf2/HO-1 axis	Regulates ferroptosis-mediated disruption of the Nrf2/HO-1 axis (Nrf2↓, HO-1↓, SLC7A11↓, GPX4↓); decreased GSH/GSSG ratio, enhances ROS formation and apoptosis as a cisplatin sensitizing agent	Lou et al. (2021)
*Morus alba* L., *Datura metel* L., and other 6 herbs	Kaempferol	A549, H460 cells	Nrf2 and miR-340	Suppresses the expression of GST, NQO1 and HO1 through downregulating Nrf2; upregulates miR-340 and PTEN; induces apoptosis and autophagy (cyclinD1↓, Bcl-2↓, Bax↑, Caspase-3↑, Caspase-9↑)	Han et al. (2018), Fouzder et al. (2021)
*Morus alba* L., *Datura metel* L., and other 6 herbs	Quercetin	Radiation-resistant NSCLC cell lines	MiR-16-5p/WEE1 axis	Increases the expression of miR-16-5p to target WEE1; enhances the radiosensitivity of NSCLC cells	Wang et al. (2020d)
*Morus alba* L., *Datura metel* L., and other 6 herbs	Quercetin	A549, H441 and H1975 *in vitro* and A549 *in vivo* models	Aurora B	Suppresses CT45A2 gene transcription by inhibiting TCF4 and β-catenin; reduces the growth of lung cancer cells	Xingyu et al. (2016)
*Eriobotrya japonica* (Thunb.) Lindl.	Hesperidin	A549, NCI-H358, H460 cells	FGF/NF-κB and miR-132/ZEB2 signaling pathway	Suppresses the expression of FGF and NF-κB and enhances apoptosis-related nucleosomal enrichment factor; upregulates miR-132 which inhibits the expression of ZEB2; anti-proliferation, apoptosis; induces cell death (Annexin V, Caspase-3, JC-1)	Birsu Cincin et al. (2015), Tan et al. (2020)
*Eriobotrya japonica* (Thunb.) Lindl., *Aster tataricus* L.f.	Luteolin	A549, H1975, and H460 cells	miR-34a-5p, Src/FAK	Inhibits cell proliferation and induces apoptosis via miR-34a-5p targeting MDM4; diminishes the p-FAK, p-Src, Rac1, Cdc42, and RhoA	Jiang et al. (2018), Masraksa et al. (2020)

**FIGURE 2:**
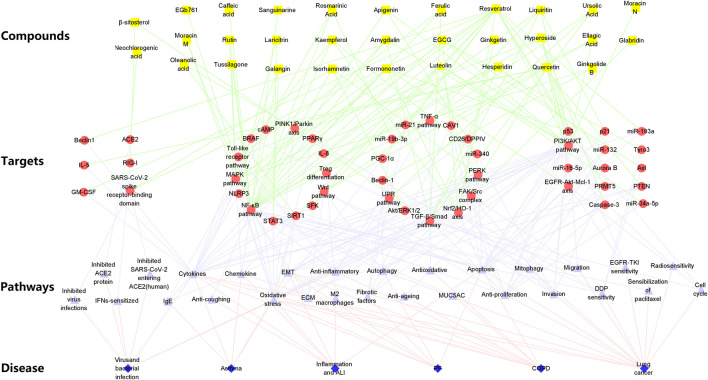
Network of bioactive compounds, targets, pathways, and six main respiratory diseases.

Collectively, the network of bioactive compounds, targets, signal pathways, and different pulmonary diseases is visualized in Figure 2. These bioactive compounds, such as isorhamnetin, formononetin, resveratrol, and galangin are active substances of types of saponins, flavonoids, and alkaloids, which can regulate different key targets (NF-κB, PI3K/Akt, Nrf-2, NLRP3) to regulate cytokine production, oxidative stress or chemotherapy sensitivity against a series of lung-related diseases.

## Discussion

**FIGURE 3:**
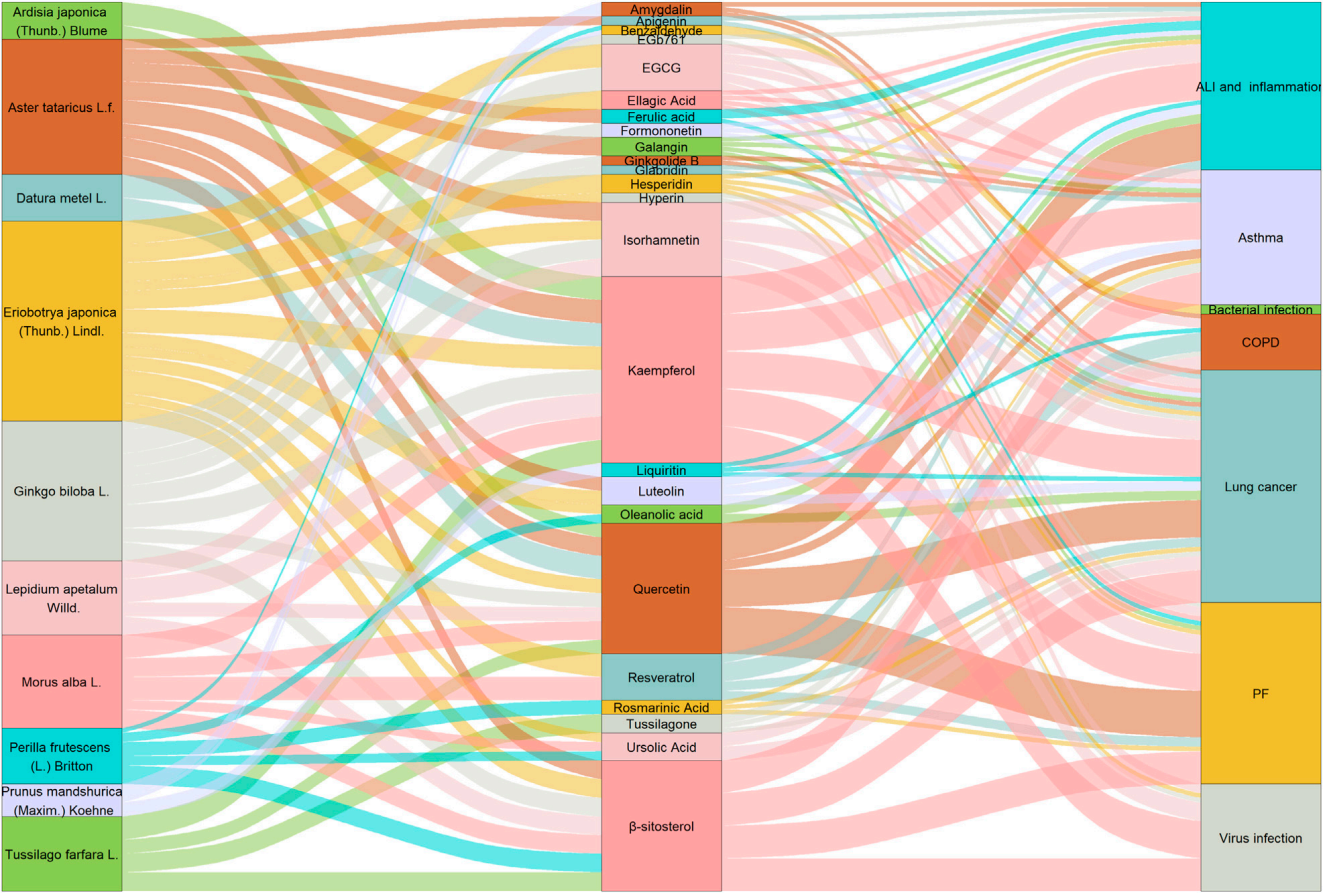
Network of different herbs, main bioactive compounds, and a series of lung-related diseases.

According to the theories of TCM and commonly used Chinese medicines in the clinical application against respiratory diseases, this review summarizes the pharmacological effects and molecular mechanisms of 31 active compounds of 10 Chinese herbal medicines in six main lung-related diseases, including pulmonary infection, ALI, PF, COPD, asthma, and lung cancer. Many studies have demonstrated that bioactive compounds can ameliorate bacterial, virus, and LPS-induced pulmonary infection by targeting the NF-κB, MAPK, Nrf2/HO-1, and NLRP3 pathways, reducing the release of cytokines and chemokines, and suppressing inflammation by pathological reaction, oxidative stress, and ROS production. *Eriobotrya japonica* (Thunb.) Lindl. and its compounds (EGCG, isorhamnetin, hesperidin, hyperoside, kaempferol, β-sitosterol) may be considered an effective Chinese herbal medicine for the treatment of viral infections. Flavonoids, including apigenin, galangin, isorhamnetin, rutin, moracin M, amygdalin, hesperidin, quercetin, formononetin, luteolin, and liquiritin, exhibit good bioactivity against ALI. As for inflammation, bioactive compounds from *Aster tataricus* L.f*.* and *Eriobotrya japonica (Thunb.) Lindl*. have potential anti-inflammatory activity, suggesting that apigenin, quercetin, luteolin, and isorhamnetin are effective anti-inflammatory compounds. In the studies of COPD, bioactive compounds have mainly attenuated cigarette smoke-induced emphysema, airway remodeling, and inflammation through the NF-κB, MAPK, and TGF-β/Smad pathways, and resveratrol is one of the important and effective bioactive substances against COPD. Multiple components, including β-sitosterol, ferulic acid, quercetin, hesperidin, EGb761, and resveratrol, are directly or indirectly related to TGF-β/Smad, which is a crucial target for PF. These components can effectively suppress biological process of EMT and ECM. In asthma, rosmarinic acid, tussilagone, formononetin, and galangin targeting the MAPK and NF-κB pathways to reduce OVA-specific IgE, and ameliorate airway hyperplasia and hypertrophy. Importantly, these active components such as organic acids and flavonoids can inhibit the proliferation and migration of lung cancer and increase its sensitivity to radiotherapy and chemotherapy. Hyperoside, resveratrol, glabridin, luteolin, and kaempferol are considered potential candidates for the treatment of lung cancer based on a large number of studies. Collectively, ECCG, kaempferol, isorhamnetin, quercetin, and β-sitosterol are important bioactive compounds for prevention and treatment of ALI, PF, and lung cancer. Taken together, multiple bioactive compounds from the 10 different herbs have potential therapeutic effects against respiratory diseases by regulating various molecular pathways (Figures 2, 3).

However, in the studies of different bioactive compounds on respiratory diseases, three important aspects should be considered. Firstly, only key and common active ingredients in each herb are summarized, which might not be fully representative of the herb. More active components should be further identified to explore their pharmacological effects against respiratory diseases. Secondly, multiple compounds In a herb can target similar or different signaling pathways to play the potential roles in those diseases. The network of various targets of different components might be used to explain the combined effect of the formula. Thirdly, different signaling pathways or pathological procedures in infection, inflammation, COPD, or lung cancer are potential targets for these active ingredients. However, the potential targets and the binding role of these active compounds still remain unclear. It should be a future direction for most researchers to confirm specific targets of those potential drug candidates using multiple modern techniques. Finally, the quantitative analysis for the biological activity, toxicity and selectivity of 31 bioactive compounds should be performed in a kind of respiratory diseases to predict the promising candidates for drug development using systematic review and meta-analysis. Overall, this review provides novel perspectives on the preclinical study and clinical application of herbal medicines and their bioactive compounds against respiratory diseases.

## Conclusion

In summary, 10 Chinese herbal medicines were selected based on the theories of TCM and high-frequency use of Chinese medicines in clinical application. The pharmacological effects and molecular mechanisms of 31 bioactive compounds from these 10 Chinese herbs in infection, ALI, PF, COPD, asthma, and lung cancer were summarized. The bioactive compounds, such as epigallocatechin-3-gallate, kaempferol, isorhamnetin, quercetin, and β-sitosterol, can mainly regulate the NF-κB, Nrf2/HO-1, NLRP3, TGF-β/Smad, MAPK, and PI3K/Akt/mTOR pathways to inhibit infection, inflammation, extracellular matrix deposition, and tumor growth in a series of lung-related diseases. This review provides novel perspectives on the preclinical study and clinical application of Chinese herbal medicines and their bioactive compounds against respiratory diseases.
